# Multiplex droplet digital PCR for the detection and quantitation of *Streptococcus pneumoniae*, *Mycoplasma pneumoniae*, and *Haemophilus influenzae*

**DOI:** 10.3389/fcimb.2025.1548492

**Published:** 2025-06-20

**Authors:** Yaling Cao, Junwen Wang, Yitong Jiang, Ling Xu, Yuan Tian, Zihao Fan, Zhenzhen Pan, Yinkang Mo, Xianru Zhu, Xiangying Zhang, Jing Huang, Feng Ren

**Affiliations:** ^1^ Beijing Institute of Hepatology/Beijing Youan Hospital, Capital Medical University, Beijing, China; ^2^ Department of Clinical Laboratory, Chui Yang Liu Hospital, Beijing, China; ^3^ Yanjing Medical College, Capital Medical University, Beijing, China; ^4^ Peking Union Medical College Hospital, Chinese Academy of Medical Sciences & Peking Union Medical College, Department of Infection Control, Beijing, China

**Keywords:** *Streptococcus pneumoniae*, *Mycoplasma pneumoniae*, *Haemophilus influenzae*, ddPCR, molecular diagnosis

## Abstract

**Background:**

Lower respiratory tract infection is one of the major causes of disease and death worldwide. *Streptococcus pneumoniae*, *Mycoplasma pneumoniae*, and *Haemophilus influenzae* are important pathogens responsible for lower respiratory tract infection. Here, we established a multiplex droplet digital polymerase chain reaction (ddPCR) method for the simultaneous detection of *S. pneumoniae*, *M. pneumoniae* and *H. influenzae* DNA.

**Methods:**

Specific primers and probes were designed for ddPCR. The sensitivity and specificity of the ddPCR assay were evaluated using standard strains, positive samples and 26 common pathogenic bacteria. One hundred and sixty-seven clinical samples were collected and tested via ddPCR, qPCR, bacterial culture and microfluidic chip technology.

**Results:**

The limits of detection (LoDs) of ddPCR were 2.5, 2.8 and 2.0 copies/μL for *S. pneumoniae*, *M. pneumoniae* and *H. influenzae*, respectively, which were approximately tenfold lower than the LoDs of qPCR. For 167 clinical samples, the positivity rates of ddPCR and microfluidic chip for *S. pneumoniae* and *M. pneumoniae* were 27.5% and 22.8%, respectively, which were higher than those of qPCR 25.7% and 21.6%. The positive rate of *H. influenzae* detection via ddPCR and microfluidic chip method was 29.9%, which was higher than that of qPCR (28.7%). The clinical sensitivity for *S. pneumoniae*, *M. pneumoniae* and *H. influenzae* improved from 97.4%, 94.7% and 95.1% for qPCR to 100% for ddPCR. Moreover, ddPCR showed less inhibition by the inhibitor in respiratory specimens than qPCR.

**Conclusion:**

The multiplex ddPCR assay established in this study can accurately detect *S. pneumoniae*, *M. pneumoniae* and *H. influenzae* DNA and can be used as an auxiliary tool for the clinical identification of pathogens and guidance of antibiotic therapy.

## Introduction

Lower respiratory tract infection is a common clinical disease that can cause symptoms such as sore throat, headache, fever, muscle aches, nausea and vomiting and can lead to pneumonia, otitis media and other diseases, resulting in high morbidity and mortality. Community-acquired pneumonia (CAP) is an infectious disease resulting in inflammation of the lung parenchyma caused by a variety of microorganisms, such as bacteria, viruses, and mycoplasma. As one of the most prevalent lower respiratory tract infections, it is contracted outside hospitals or other common medical institutions and is one of the most common lower respiratory diseases in the clinic (GBD 2017 Causes of Death Collaborators, 2018; Rider and Frazee, 2018; Claassen-Weitz et al., 2021; Musungu et al., 2024). CAP causes high morbidity and mortality in adults in developed countries and is one of the leading causes of child mortality in developing countries (Zar et al., 2016; Pneumonia Etiology Research for Child Health (PERCH) Study Group, 2019; Meyer Sauteur, 2024). In the 2010 Global Burden of Disease Study, lower respiratory infections, including pneumonia, were ranked as the fourth leading cause of death worldwide (Blasi et al., 2012; Lozano et al., 2012; Cillóniz et al., 2020). Although the widespread implementation of immunization programs for *Haemophilus influenzae* type b and pneumococcal conjugate vaccines has led to a decline in mortality from CAP caused by bacterial infections, bacteria play a momentous role in pneumonia, and evidence has shown that viral–bacterial coinfections remain common (Ruuskanen et al., 2011; Li et al., 2020; Feldman and Anderson, 2021). A CAP infection surveillance study from China revealed that the three most prevalent bacterial pathogens were *Streptococcus pneumoniae*, *Haemophilus influenzae*, and *Mycoplasma pneumoniae* in children and adolescents and *S. pneumoniae*, *Klebsiella pneumoniae*, and *Pseudomonas aeruginosa* in adults and elderly individuals (Liu et al., 2023). Thus, the rapid and accurate detection of bacterial pathogens is helpful for providing timely clinical antibiotic treatment.

Currently, pathogen cultures, time–flight mass spectrometry and biochemical indicators are mainly used to identify infectious pathogens in the clinic. However, *S. pneumoniae*, *M. pneumoniae* and *H. influenzae* are difficult to isolate and culture because of the stringent environmental conditions and large amount of time required for culture, resulting in a low detection rate in the clinic (Daxboeck et al., 2003; Torigoe et al., 2007). Molecular detection techniques such as qPCR are rapid molecular diagnostic tools applied in the clinic to detect a wide range of microorganisms (Loens and Ieven, 2016; Sunaga et al., 2020; Zhao et al., 2020). However, for samples with low target concentrations, qPCR does not show ideal sensitivity. Therefore, a new molecular assay for the rapid and accurate detection of *S. pneumoniae*, *M. pneumoniae* and *H. influenzae* is needed.

Droplet digital PCR (ddPCR) is an absolute quantitative analytical technique based on the single-molecule PCR method to count nucleic acid molecules (Vogelstein and Kinzler, 1999). Mechanically speaking, a large amount of diluted nucleic acid solution is dispersed into microreactors or microdroplets on a chip via microfluidics or microtitration, and the number of nucleic acid templates per reactor is less than or equal to 1. Afterward, each individual partition acts as a separate microreactor, and the target gene is amplified via PCR cycles. Eventually, the nucleic acid concentration of the original solution can be deduced on the basis of the relative proportions and the volumes of the reactors. In recent years, ddPCR has been widely used, such as for the detection of the absolute viral load from various clinical samples and the analysis of gene copy number variation, gene expression, and genome edit detection (White et al., 2009; Postel et al., 2018). Therefore, this study aimed to establish a multiplex ddPCR assay for the simultaneous detection of *S. pneumoniae*, *M. pneumoniae* and *H. influenzae* to aid in the diagnosis of CAP-associated pathogens.

## Materials and methods

### Study design

**Figure 1:**
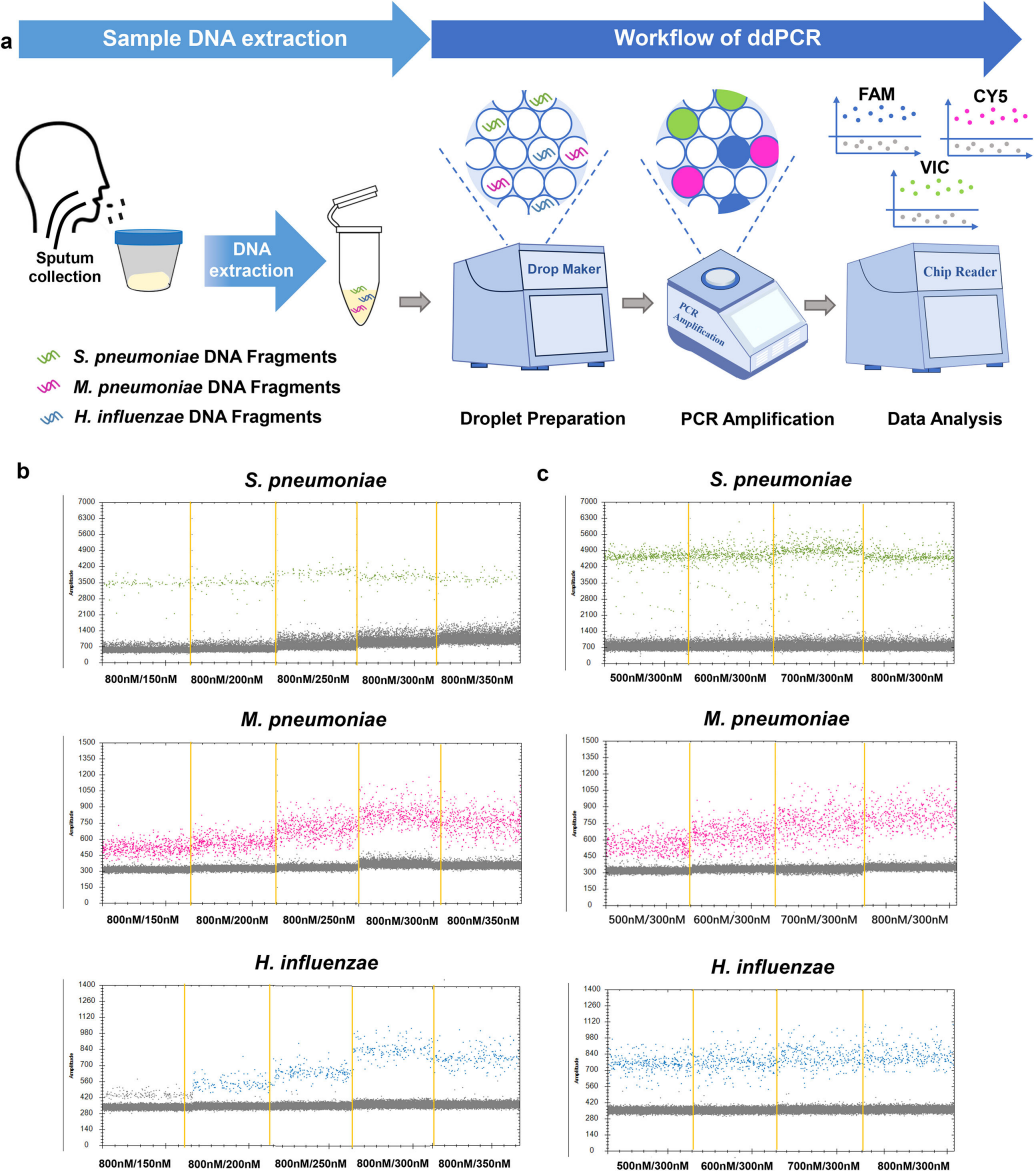
Establishment and optimization of the multiplex ddPCR assay for *S. pneumoniae, M. pneumoniae and H. influenzae* detection. **(a)** Schematic of the multiplex ddPCR assay. The detection procedure consists of a two-step method: nucleic acid extraction followed by ddPCR detection. After extracting nucleic acids from the samples, the process continues with droplet preparation, PCR amplification. Finally, the results were analyzed by observing the fluorescence droplet plot in the FAM, VIC and CY5 channels via a QX200 reader. The droplets that were positive are indicated in green (*S. pneumoniae*), pink (*M. pneumoniae*) and blue (*H. influenzae*), the negative droplets appeared gray. **(b, c)** Optimal concentrations of primers and probes were determined by the results of detection of mixing positive samples. Screening Principle: The optimal probe and primer concentrations were selected according to whether the positive droplets were distributed centrally and whether they could be differentiated from the negative droplets efficiently. The experiment was repeated three times for each concentration (means ± SD).

In this study, we constructed a multiplex ddPCR assay for the simultaneous detection of *S. pneumoniae*, *M. pneumoniae* and *H. influenzae*, and validated the clinical application. The multiplex ddPCR detection process can be divided into three parts. First, collect clinical samples for DNA extraction, followed by droplet preparation, PCR amplification. Finally, measure the fluorescence intensities in the (FAM/VIC/CY5) channels for data analysis (**Figure 1a**).

### Standard strains and clinical specimen collection and cultivation

The standard strains used were *S. pneumoniae* ATCC 49619, and *H. influenzae* ATCC 49247, and single colonies were obtained after performing bacterial culture. *M. pneumoniae* positive DNA samples were identified via *Mycoplasma pneumoniae* and *Chlamydia pneumoniae* nucleic acid detection kit (real-time PCR, Shanghai ZJ Bio-Tech Co.Ltd.). Meanwhile, *M. pneumoniae* ATCC 15531 was cultured, and bacterial genomic DNA was extracted after confirming a positive culture using phenol red pH indicator to determine the LoD of the ddPCR method for *M. pneumoniae* detection. Genomic DNA extracted from these standard reference strains or positive samples was quantified by ddPCR and subsequently used for methodological evaluation. We collected sputum and bronchoalveolar lavage (BAL) samples from 167 patients with lower respiratory tract symptoms (such as bronchitis, tracheitis, and pneumonia, presenting with sore throat, cough, and fever) at Beijing Chuiyangliu Hospital (Between January 2024 and November 2024). Sample types included 157 sputum and 10 BAL specimens. Patient characteristics are summarized in **Supplementary Table 1**. We excluded samples with insufficient volume (<300 μL). We also must exclude unqualified samples. We consider a sample qualified if there are more than 25 white blood cells and less than 10 (or 10-25) epithelial cells per low-power field. After sample collection, bacterial culture was performed within 2 hours, followed by identification using Matrix-Assisted Laser Desorption/Ionization Time of Flight Mass Spectrometry (MALDI-TOF MS). DNA extraction was also performed and samples were stored at -20°C if DNA was not extracted in time, during which repeated freezing and thawing were avoided. After the DNA was extracted, the samples were tested via ddPCR, qPCR, microfluidic chip technology and microbial culture.

### DNA extraction

A bacterial DNA extraction kit (Beijing Tiangen Biochemical Technology Co., Ltd.) was used for the extraction of DNA from the bacterial strains. A sputum DNA extraction kit (China Ying li Baio Biotechnology Co., Ltd.) and a universal genomic DNA extraction kit (Beijing Tiangen Biochemical Technology Co., Ltd.) were used for the extraction of DNA from sputum samples and BAL samples. A 300 μL sample volume was used for DNA extraction. The extracted DNA was stored in a refrigerator at -20°C.

### Design and screening of probe, primers and plasmid synthesis

In this study, we selected the N-acetylmuramoyl-L-alanine amidase gene (*lytA*) of *S. pneumoniae* (Reference sequence GenBank accession: AP018938.1), the community-acquired respiratory distress syndrome toxin gene (*CARDS*) of *M. pneumoniae* (Reference sequence GenBank accession: LR214945.1) and the outer membrane protein P6 gene (*ompP6*) of *H. influenzae* (Reference sequence GenBank accession: KC332053.1) as target sequences (Reference sequence details are provided in the **Supplementary Material**). These targets are often used to detect the corresponding pathogens in previous studies because of their specificity (Zhou et al., 2023, Qiu et al., 2023, Cheng et al., 2022). Three pairs of primers and one probe were designed for each target. Three hundred to 500 bp of each target sequence was selected for plasmid construction, and the sequences were synthesized by Shanghai Sangyo (**Supplementary Table 2**). DNA copy number (copies/µL)=[6.02×10^23^×genomic DNA concentration (ng/µL)×10^−9^]/[genomic DNA length (nt)×660] (Yang et al., 2021). The plasmids were used for qPCR detection, and the best primers were selected according to the threshold cycle (CT) value.

### System optimization

Optimal concentrations of primers and probes were determined by the results of detection of mixing positive samples. The primer concentration was determined to be 800 nM, and the optimal probe concentration was selected from the following gradient (150 nM, 200 nM, 250 nM, 300 nM, 350 nM) based on two criteria: (1) whether the positive droplets were distributed centrally, and (2) whether they could be differentiated from the negative droplets efficiently. Then, the optimal primer concentrations were determined immediately after the probe concentrations were determined. The concentrations of 500 nM, 600 nM, 700 nM and 800 nM were selected for screening. The screening criteria are consistent with those described previously.

### ddPCR workflow

The instrument used for these experiments was Xin Yi Biotechnology Co. The process of ddPCR mainly consists of three parts: droplet preparation, PCR amplification, and result analysis. The total volume of the reaction was 30 μL, including 7.5 μL of 4× hypermixed probe (no dUTP), 2.4 μL of 10 mM F/R, 0.9 μL of probe, 0.4 μL of DNase/RNase-free water, DNA template 5μL, followed by the addition of 180 μL of droplet-generation oil and a droplet generator was used to convert the reaction mixture into droplets. The next step is the amplification process. The reaction conditions were as follows: thermal cycling, with the reaction conditions of 95°C for 10 min, followed by 94°C for 30 s (denaturation) and 60°C for 1 min (annealing) for 39 cycles, followed by an infinite hold of 4 degrees. Finally, observe the fluorescence analysis results. The tubes containing the products were transferred and read in the FAM, VIC and CY5 channels via a Fluorescence reader (Xin Yi Biotechnology Co). The FAM, VIC, and CY5 channels represent *S. pneumoniae*, *M. pneumoniae* and *H. influenzae* detection results, respectively.

### qPCR analysis

The primers and probes used for ddPCR were used for the establishment of the PCR detection system, and qPCR was performed via an LightCycler­^®^ 480 Instrument II. The total volume of the reaction was 20 μL. The reaction system included 10 μL of Fast Advanced Master Mix (Thermo Fisher Scientific, Waltham, MA, USA), 0.5 μL of the 10 nM primer and probe mixture, 2.5 μL of DNase/RNase-free water and 3 μL of DNA template, and the reaction conditions were as follows: denaturation at 94°C for 3 min, denaturation at 94°C for 30 s, annealing at 58°C for 45 s, and 35 cycles.

### Determination of dynamic ranges and limits of detection of ddPCR and qPCR

The dynamic range of the ddPCR and PCR methods were determined via gradient dilutions of *S. pneumoniae, M. pneumoniae and H. influenzae* standard strains as templates, with ddPCR template concentrations of 1 copy/μL - 10^5^ copies/μL and qPCR template concentrations of 1 copy/μL-10^6^ copies/μL, respectively. The concentration range with good linear relationship was the dynamic monitoring range. For Limit of Quantification (LoQ) determination, we performed replicate analyses at copy number concentrations approximating the LoD for both methods. For ddPCR, we performed replicate testing at two concentrations (10 copies/μL and 1 copy/μL), while for qPCR, we tested replicates at 100 copies/μL and 10 copies/μL. The lowest concentration achieving a coefficient of variation (CV) ≤20% was defined as the LoQ. Similarly, the LoDs were determined via gradient dilutions of *S. pneumoniae, M. pneumoniae and H. influenzae* standard strains as templates, with ddPCR template concentrations of 10 copies/μL, 5 copies/μL, 1 copy/μL, 0.5 copies/μL, and 0.1 copies/μL and qPCR template concentrations of 10^3^ copies/μL, 10^2^ copies/μL, 10 copies/μL, 1 copy/μL, respectively. Different template concentrations were tested to determine the LoD as well as to perform a regression analysis with 95% reproducibility probability, which is a commonly used regression analysis model for analyzing the reliability of molecular detection methods (Liu et al., 2020).

### Specificity of the ddPCR assay

To evaluate the specificity of the ddPCR assay, we collected clinical reference strains and isolates of non-*Mycoplasma pneumoniae*, *Mycoplasma pneumoniae*, and *Haemophilus influenzae*. The names and numbers of specific strains are given in **Table 1**. After culturing the strains, bacterial DNA was extracted using a genomic DNA extraction kit (Beijing Tiangen Biochemical Technology Co., Ltd.) with a concentration of approximately 10^5^ copies/μL. The specificity of the ddPCR method was then assessed by comparing the detected copy numbers between non-target and target bacterial strains. *S. pneumoniae* reference strains, *M. pneumoniae* positive samples, and *H. influenzae* reference strains were used as positive controls (PCs), and nuclease-free water was used as a negative control (NC).

**Table 1:** Bacterial strains used in this study.

Strain	Type (Strain ID)	Source of strains	Number of strains used	ddPCR test
*Haemophilus influenzae*	Reference strain (ATCC 49247)	Chui Yang Liu Hospital	1	P
*Streptococcus pneumoniae*	Reference strain (ATCC 49619)	Chui Yang Liu Hospital	1	P
*Mycoplasma pneumoniae*	Reference strain (ATCC 15531)	Chui Yang Liu Hospital	1	P
*Mycoplasma pneumoniae*	Clinical samples DNA	Chui Yang Liu Hospital	3	P
*Klebsiella pneumoniae*	Reference strain (ATCC 700603)	Beijing Youan Hospital	1	N
*Pseudomonas aeruginosa*	Reference strain (ATCC 27853)	Chui Yang Liu Hospital	1	N
*Staphylococcus aureus*	Reference strain (ATCC 29213)	Beijing Youan Hospital	1	N
*Acinetobacter baumannii*	Clinical strain	Beijing Youan Hospital	2	N
*Mycobacterium tuberculosis*	Reference strain (H37Rv)	Chui Yang Liu Hospital	1	N
*Corynebacterium pseudodiphtheriticum*	Clinical strain	Chui Yang Liu Hospital	1	N
*Haemophilus parainfluenzae*	Clinical strain	Chui Yang Liu Hospital	1	N
*Haemophilus haemolyticus*	Clinical strain	Chui Yang Liu Hospital	1	N
*Haemophilus parahaemolyticus*	Clinical strain	Chui Yang Liu Hospital	1	N
*Escherichia coli*	Reference strain (ATCC 25922)	Beijing Youan Hospital	1	N
*Candida albicans*	Clinical strain	Chui Yang Liu Hospital	1	N
*Stenotrophomonas maltophilia*	Clinical strain	Chui Yang Liu Hospital	1	N
*Streptococcus pyogenes*	Clinical strain	Chui Yang Liu Hospital	1	N
*Streptococcus salivarius*	Clinical strain	Chui Yang Liu Hospital	1	N
*Streptococcus mitis*	Clinical strain	Chui Yang Liu Hospital	1	N
*Streptococcus agalactiae*	Clinical strain	Chui Yang Liu Hospital	1	N
*Streptococcus oralis*	Clinical strain	Chui Yang Liu Hospital	1	N
*Streptococcus suis*	Clinical strain	Chui Yang Liu Hospital	1	N
*Streptococcus mutans*	Clinical strain	Chui Yang Liu Hospital	1	N
*Streptococcus sanguinis*	Clinical strain	Chui Yang Liu Hospital	1	N
*Mycoplasma genitalium*	Clinical strain	Chui Yang Liu Hospital	1	N
*Mycoplasma primatum*	Clinical strain	Chui Yang Liu Hospital	1	N
*Clostridium difficile*	Reference strain (ATCC BAA-1803)	Chui Yang Liu Hospital	1	N
*Listeria monocytogenes*	Clinical strain	Chui Yang Liu Hospital	1	N
*Mycoplasma urealyticum*	Clinical strain	Chui Yang Liu Hospital	1	N
*Mycoplasma hominis*	Clinical strain	Chui Yang Liu Hospital	1	N

The clinical strains were laboratory-stored strains obtained from corresponding positive clinical samples.

### Repeatability of the ddPCR assay

The repeatability of the ddPCR assay was evaluated via intra-assay and interassay methods using *S. pneumoniae*, *M. pneumoniae* and *H. influenzae* mixed positive samples. Two mixed *S. pneumoniae, M. pneumoniae and H. influenzae* positive samples were used in the same experiment for six replicates, and three separate ddPCR tests were subsequently conducted.

### Evaluation of ddPCR and qPCR inhibition by respiratory specimens

To determine the inhibition effect of respiratory specimens on PCR amplification, equal amounts of plasmid DNA were added to samples containing different amounts (2μL to 8μL) of extract of respiratory specimen to prepare spiked samples. Simultaneously prepare control samples (2μL to 8μL of DNase-free water and plasmid at the same concentration). Calculate and compare the inhibition rates using the ddPCR and qPCR results (CT values converted to copy numbers) and the control sample results (copy numbers). Then calculate the inhibition rate (%) using the formula: [(Control copy number – ddPCR/qPCR copy number)/Control copy number×100] to evaluate the impact of respiratory specimens on the detection of ddPCR and qPCR.

### Statistical analysis

According to the principle of ddPCR, each sample under test was divided into 10000 reaction units. Each tiny droplet contained one or more copies of the template, and microdroplets appeared when there was a target product fluorescent signal. The reaction unit was marked as “1”. After PCR amplification, according to the Poisson probability distribution formula, the ddPCR number data were analyzed via specific software to calculate the target concentration. Prism (GraphPad, La Jolla, CA) 8.00 software was used to perform linear regression, and probabilistic regression analysis was performed with MedCalc 19.0.4 software (MedCalc, Ostend, Belgium).

All methods were performed in accordance with the relevant guidelines and regulations.

## Results

### Establishment and optimization of the multiplex ddPCR assay for *S. pneumoniae, M. pneumoniae and H. influenzae*

Multiple pairs of primers were designed for *S. pneumoniae, M. pneumoniae and H. influenzae* target sequences. By comparing the CT values of different primers in single qPCR, the best primers for *S. pneumoniae, M. pneumoniae and H. influenzae* were selected for subsequent ddPCR and qPCR, as shown in **Supplementary Table 2**. The optimal primer pairs are *S. pneumoniae* -F1/R1, *M. pneumoniae*-F2/R2, and *H. influenzae* -F1/R1 (for *S. pneumoniae, M. pneumoniae and H. influenzae*, respectively, **Supplementary Figure 1**). Optimal concentrations of primers and probes were determined by the results of detection of mixing positive samples. The system was optimized by observing the fluorescent droplets results for the three fluorescence channels (aggregation of fluorescent droplets and the dispersion distance of positive droplets from negative droplets) with different primer and probe concentrations. The final primer concentration of 800 nM and probe concentration of 300 nM were used for subsequent experiments (**Figures 1b, c**).

### Sensitivity and dynamic range of ddPCR and qPCR

**Figure 2:**
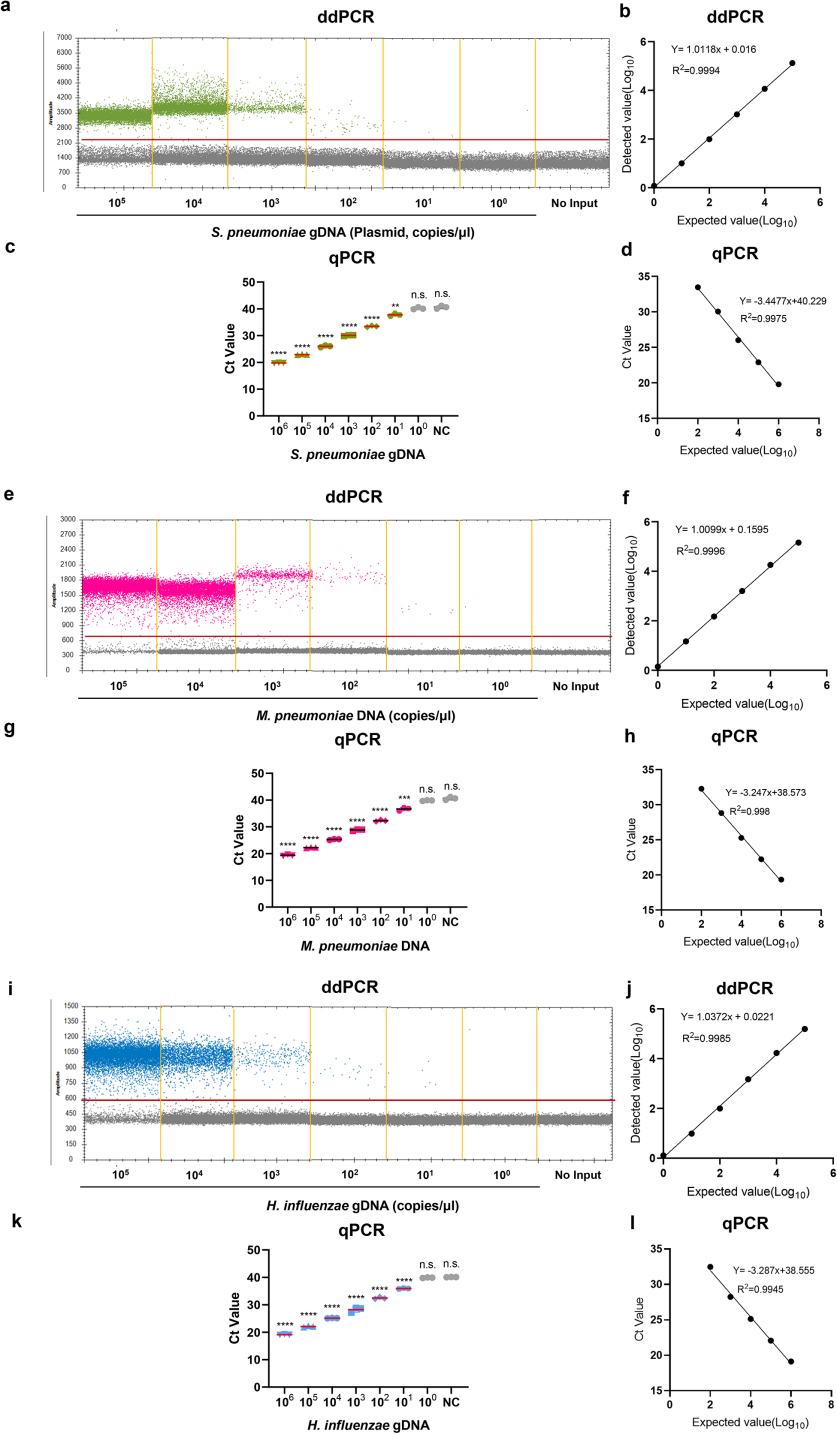
The dynamic range of the ddPCR assay to detect *S. pneumoniae, M. pneumoniae and H. influenzae* DNA. **(a, e, i)** The 10-fold serial dilution of *S. pneumoniae* gDNA, *M. pneumoniae* DNA, *H. influenzae* gDNA were detected by the ddPCR; the green points, the pink points and the blue points represent the positive signal; Correlation analysis to determine the dynamic detection range. **(b, f, j)** The expected values (converted to log10) of *S. pneumoniae* gDNA, *M. pneumoniae* DNA, *H. influenzae* gDNA were plotted on the Y axis and ddPCR detected values (converted to log10) on the X axis to perform linear analysis. Data are representative of at least three repeated experiments for different concentrations of template DNA. **(c, g, k)** The 10-fold serial dilution of *S. pneumoniae* gDNA, *M. pneumoniae* DNA, *H. influenzae* gDNA were detected by the qPCR; **(d, h, l)** The expected values (converted to log10) of *S. pneumoniae* gDNA, *M. pneumoniae* DNA, *H. influenzae* gDNA were plotted on the Y axis and qPCR detected values (converted to log10) on the X axis to perform linear analysis. DNase/RNase-free water was used as the negative control. The experiment was repeated three times (means ± SD). **P < 0.01, ***P < 0.001, ****P < 0.0001. "n.s." indicates no statistically significant difference.

To compare the dynamic range of ddPCR and qPCR, multiplex ddPCR and multiplex qPCR were performed with serial dilutions of reference strain DNA (*S. pneumoniae*, *H. influenzae*) and *M. pneumoniae* positive sample DNA for each target, and the results revealed that the dynamic range of ddPCR was 1–10^5^ copies/μL for *S. pneumoniae, M. pneumoniae and H. influenzae* DNA. Subsequently, we determined that the LOQ of ddPCR for *S. pneumoniae, M. pneumoniae and H. influenzae* DNA detection was 10 copies/reaction. Therefore, the results revealed that the reliable range of ddPCR was 10–10^5^ copies/μL for *S. pneumoniae, M. pneumoniae and H. influenzae* DNA (**Figures 2a, e, i**). The linear regression analysis revealed a good linear relationship between the detected and expected values of ddPCR, with R^2^ values of 0.9994, 0.9996, and 0.9985 (**Figures 2b, f, j**). Similarly, the results revealed the reportable range of qPCR was 10^2–^10^6^ copies/μL (**Figures 2c, g, k**). The results showed that excellent linear correlation between the detected value and expected value, with R^2^ values of 0.9975, 0.998, and 0.9945 for the *S. pneumoniae, M. pneumoniae and H. influenzae* primer–probe sets, respectively (**Figures 2d, h, l**). Subsequently, we have supplemented both ddPCR and qPCR detection results using serially diluted *M. pneumoniae* standard strains as templates. The results demonstrate that ddPCR exhibited an effective detection reliable range of 10-10⁵ copies/μL, while qPCR showed a range of 10²-10⁶ copies/μL (**Supplementary Figure 2**). The above results revealed that ddPCR has a lower minimum detectable range than does qPCR.

**Figure 3:**
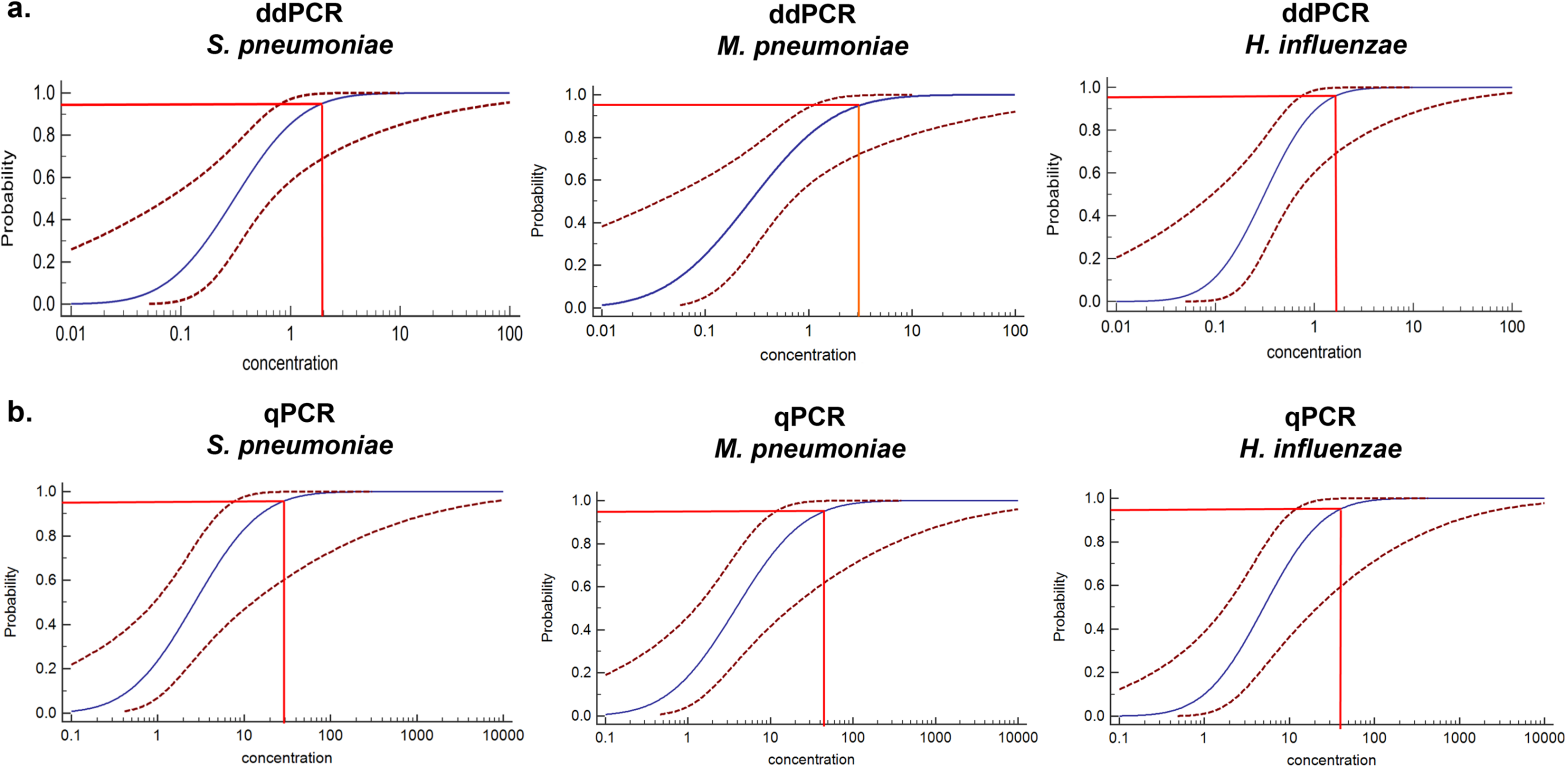
Determining the LoD of the ddPCR assay. **(a)** The LoD for *S. pneumoniae*, *M. pneumoniae*, and *H*. *influenzae* in the multiplex qPCR assay was determined using probit analysis of sigmoid curves. Repeated ddPCR assays with different concentrations of *S. pneumoniae*, *M. pneumoniae* and *H*. *influenzae* DNA were performed near the detection limits determined in the pre-experiment. The X-axis represents the expected concentration, and the Y-axis represents the proportion of positive results in the same experiment. The blue line is the probit curve, and the red dashed line are 95% confidence interval (95% CI), the experiment was repeated six times for each concentration in the same parallel reactions. **(b)** The probit analysis sigmoid curve was used to determine the LoD of the multiplex qPCR for *S. pneumoniae*, *M. pneumoniae* and *H*. *influenzae* detection. The analytical method was the same as in **(a)**.

Next, we used probit analysis with a sigmoid curve to calculate the 95% probability of reproducibility and further determined the accurate LoDs for ddPCR and qPCR via different concentrations of the reference strain DNA near concentrations close to the lower LoD, with six replicates performed for each concentration. The results revealed that the LoDs (95% probability) of the ddPCR method were 2.5 (95% CI: 1.0-87.5) copies/μL, 3.1 (95% CI: 1.1-305.8) copies/μL and 1.5 (95% CI: 0.7-38.9) copies/μL for the *S. pneumoniae, M. pneumoniae and H. influenzae* primer/probe sets, respectively (**Figure 3a**). For qPCR, the LoDs (95% probability) were 25.9 (95% CI: 7.3-6.1×10^3^) copies/μL, 44.2 (95% CI: 11.6-6.6×10^3^) copies/μL and 39.7 (95% CI: 11.9-3.1×10^3^) copies/μL, respectively (**Figure 3b**). To determine a more accurate LoDs, we performed twenty replicates for each concentration. The results revealed that the LoDs (95% probability) of the ddPCR method were 2.5 (95% CI: 1.5-6.9) copies/μL, 2.8 (95% CI: 1.7-7.8) copies/μL and 2.0 (95% CI: 1.2-5.5) copies/μL for the *S. pneumoniae*, *M. pneumoniae* and *H. influenzae* primer/probe sets, respectively (**Supplementary Figure 3a**). For qPCR, the LoDs (95% probability) were 27.6 (95% CI: 11.9-146.6) copies/μL, 41.1 (95% CI: 17.7-200.9) copies/μL and 38.8 (95% CI: 16.5-198.1) copies/μL, respectively (**Supplementary Figure 3b**). In summary, for ddPCR, when the same *S. pneumoniae, M. pneumoniae and H. influenzae* primer/probe sets were used, the low-concentration template was approximately 20 times smaller than that of qPCR (maximum). The positive threshold was determined by performing 10 replicate tests using negative controls as templates, with the threshold set at the concentration corresponding to the mean signal value plus 3 standard deviations (3SD) of the negative controls. The thresholds for the *S. pneumoniae, M. pneumoniae and H. influenzae* were 3.9, 3.5, and 3.7 copies/μL, respectively. The negative control was sputum samples without the target bacteria (**Supplementary Table 3**).

### Specificity of the ddPCR and qPCR methods

**Figure 4:**
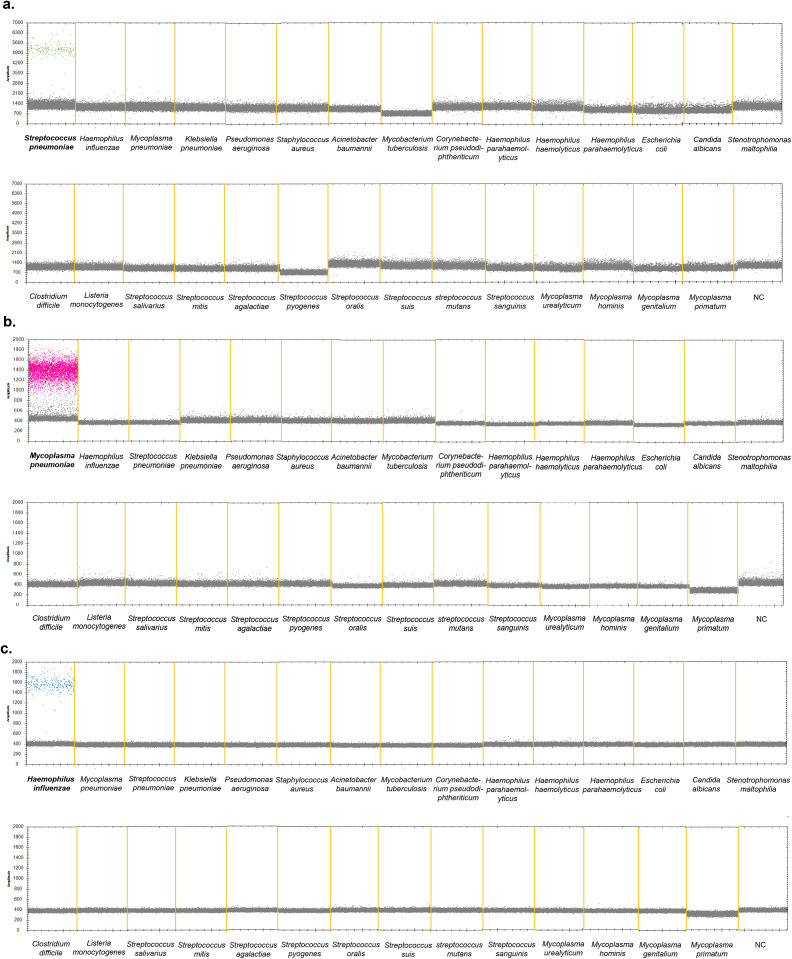
Specificity of the ddPCR assay to detect *S. pneumoniae*, *M. pneumoniae* and *H*. *influenzae*. 26 pathogenic bacteria were used to determine the specificity of the multiplex ddPCR. **(a)** VIC channel detection results. **(b)** CY5 channel detection results. **(c)** FAM channel detection results. *S. pneumoniae* (ATCC 49619), *M. pneumoniae* positive sample and *H*, *influenzae* (ATCC 49247) were used as the positive control strain. DNase-free water was used as negative control. The droplets that were positive were indicated in green (*S. pneumonia*), pink (*M. pneumoniae*) and blue (*H. influenzae*), the negative droplets appeared gray.

Twenty-six bacterial strains were collected, and DNA was extracted for ddPCR. Details regarding the bacterial strains and their corresponding quantities are listed in **Table 1**. The results showed that positive droplets were only detected in the corresponding fluorescence channels for DNA from *Streptococcus pneumoniae*, *Mycoplasma pneumoniae*, and *Haemophilus influenzae* positive strains, with copy number quantification results consistent with this observation, suggesting that the ddPCR method has good specificity (**Figures 4a–c**, **Supplementary Table 4**).

### Repeatability and reproducibility of the ddPCR assay

Six replicates of the same experiment and six separate ddPCR experiments were performed using two mixed samples of *S. pneumoniae, M. pneumoniae and H. influenzae*. The concentration of the mixed samples was set around 1–5 times the LOD value. The results revealed mean coefficients of variation (CVs) for *S. pneumoniae, M. pneumoniae and H. influenzae* DNA of 0.142, 0.221, 0.152, 0.231, 0.144, 0.250 for intraassay variation and 0.151, 0.210, 0.154, 0.207, 0.162 and 0.211 for interassay variation, respectively (**Table 2**).

**Table 2:** Repeatability and reproducibility assays of ddPCR in detection of *S. pneumoniae*, *M. pneumoniae* and *H. influenzae* DNA.

Pathogen	Intraassay variation			Interassay variation		
	Mean concentration (copies/μL)	SD	CV	Mean concentration (copies/μL)	SD	CV
*S. pneumoniae*	18.9	2.7	0.142	24.1	3.6	0.151
	2.0	0.4	0.221	2.5	0.5	0.210
*M. pneumoniae*	23.3	3.5	0.152	22.9	3.5	0.154
	2.3	2.4	0.231	2.0	0.4	0.207
*H. influenzae*	24.4	3.5	0.144	24.6	4.0	0.162
	2.2	0.6	0.250	2.3	0.5	0.211

SD, Standard Deviation; CV, Coefficient of Variation.

### Clinical sample validation

**Figure 5:**
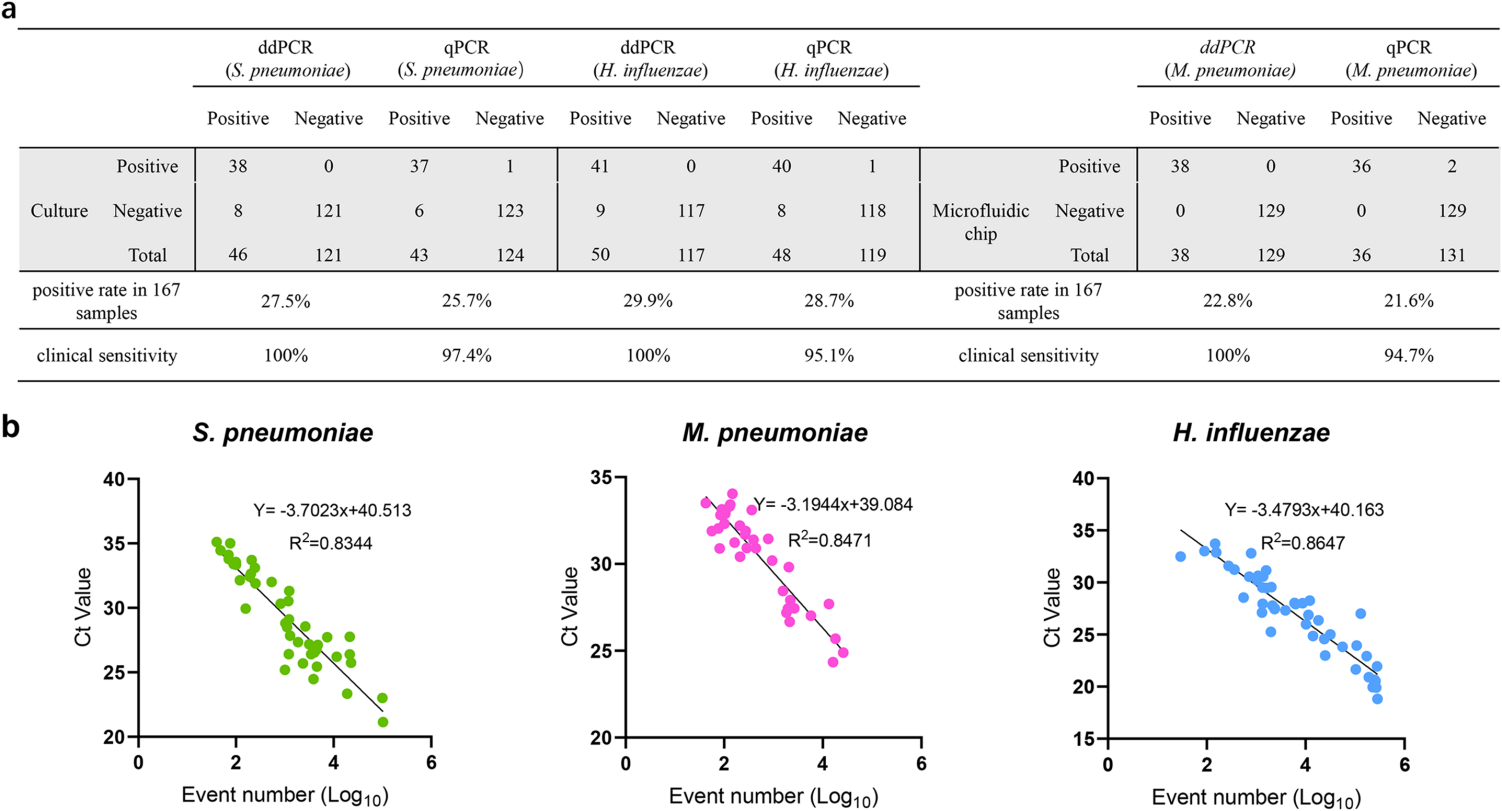
Comparison of the detection efficiency of qPCR, ddPCR and chip inspection in detection of *S. pneumoniae*, *M. pneumoniae*, *H. influenzae* in clinical samples. **(a)** The detection results of qPCR, ddPCR, bacterial culture or chip inspection in detection of *S. pneumoniae*, *M. pneumoniae*, *H. influenzae* in 167 clinical samples. **(b)** Correlation between qPCR and ddPCR. Samples that were detectable by both ddPCR and qPCR were analyzed. Event numbers for ddPCR are plotted on the x axis and the threshold cycle for qPCR on the y axis.

A total of 167 clinical samples were tested for the detection of *S. pneumoniae* and ddPCR and qPCR detected *S. pneumoniae* in 46 (27.5%) and 43 (25.7%) samples, respectively. *H. influenzae* was detected in 50 samples via ddPCR and 48 samples via qPCR, and the positive rates were 29.9% and 28.7%, respectively. Bacterial culture was used as the gold standard for the detection of *S. pneumoniae* and *H. influenzae*, and the clinical sensitivity improved from 97.4% (84.6-99.9%), 95.1% (82.2-99.1%) for qPCR to 100% (88.6-100%), 100% (89.3-100%) for ddPCR for *S. pneumoniae* and *H. influenzae*. ddPCR and qPCR detected *M. pneumoniae* in 38 (22.8%) and 36 (21.6%) samples, respectively. When microfluidic chip technology was used as the gold standard for *M. pneumoniae* detection, the clinical sensitivity for *M. pneumoniae* improved from 94.7% (80.9-99.1%) for qPCR to 100% (88.6-100%) for ddPCR. *M. pneumoniae* and *H. influenzae* were detected simultaneously in 5 samples, *S. pneumoniae* and *H. influenzae* were detected simultaneously in 3 samples. The remaining 39 samples did not contain *S. pneumoniae*, *M. pneumoniae* or *H. influenzae* DNA. For the samples with inconsistent results in *S. pneumoniae* and *M. pneumoniae* detected by ddPCR and qPCR, microfluidic chip technology was used to verify the results. Among the qPCR results, three *S. pneumoniae*, two *M. pneumoniae*, and two *H. influenzae* samples initially tested negative but were subsequently identified as positive via microfluidic chip analysis, indicating false negatives in the qPCR assay. In contrast, the ddPCR results were consistent with the microfluidic chip findings, with no false positives observed. (**Figure 5a**, **Supplementary Table 5**). Moreover, linear regression and correlation analysis were performed for all samples with positive ddPCR and qPCR results, and the results showed that the ddPCR log event number increased with increasing qPCR Ct values, with R^2^ values of 0.8344, 0.8471, and 0.8647 respectively for the detection of *S. pneumoniae, M. pneumoniae and H. influenzae* (**Figure 5b**).

### Inhibition of respiratory specimens on ddPCR and qPCR assays

**Figure 6:**
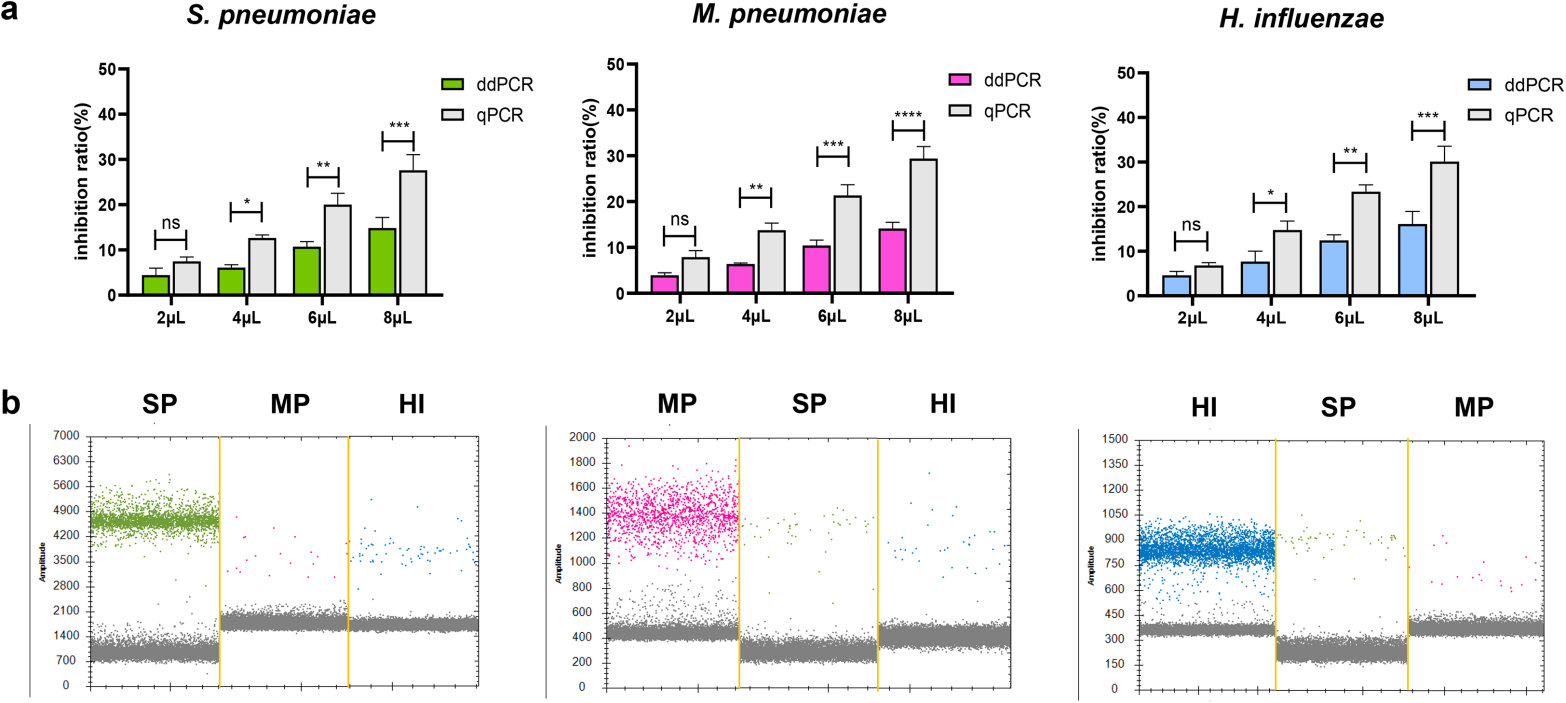
Evaluation of the interference resistance capability in ddPCR assay. **(a)** Evaluation of respiratory specimen extracts’ impact on quantitative detection of *S. pneumoniae*, *M. pneumoniae* and *H. influenzae* DNA by ddPCR and qPCR. Equal amounts of plasmid DNA were added to samples containing different amounts (2μL to 8μL) of extract of respiratory specimen to prepare spiked samples. Simultaneously prepare control samples (2μL to 8μL of DNase-free water and plasmid at the same concentration). Calculate and compare the inhibition rates using the ddPCR and qPCR results (CT values converted to copy numbers) and the control sample results (copy numbers). Inhibition rate (%) calculation formula: [(Control copy number – ddPCR/qPCR copy number)/Control copy number×100]. *P < 0.05, **P < 0.01, ***P < 0.001, ****P < 0.0001. **(b)** To observe the influence of a single high concentration pathogen on the detection of the other two pathogens in the co-infection detection. Sequentially: high-concentration samples of SP, MP, and HI. The droplets that were positive were indicated in green (SP), pink (MP) and blue (HI), the negative droplets appeared gray. SP, *S. pneumoniae*, MP, *M. pneumoniae*, HI, *H. influenzae*. "n.s." indicates no statistically significant difference.

Previous studies have shown that PCR inhibitors exist in biological specimens, which can affect the quantification of target PCR products (Poh et al., 2020). To determine the inhibition effect of respiratory specimens on ddPCR and qPCR. We mixed equal concentrations of plasmid with different volumes of extract of respiratory specimens and performed ddPCR and qPCR. We observed that both methods were affected by respiratory specimens. However, with an increase in the proportion of respiratory tract extracts, the inhibition rate of qPCR was found to be higher compared to that of ddPCR. The result showed that the inhibition rate at 6μL and 8μL for ddPCR was significantly lower than that for qPCR (**Figure 6a**). At the same time, due to the presence of co-infection in clinical detection, we tested the detection performance of two low concentrations of pathogens in the mixed samples when one of the pathogens was at high concentration, and the results showed that the ideal detection effect could be achieved (The high concentration is about 10^4^ copies/μL and the low concentration is 10^1^ copies/μL, **Figure 6b**). Additionally, we analyzed the same sample stored at 4°C for 2 hours, 24 hours, and 48 hours respectively to evaluate the impact of sample freshness on ddPCR results. The data showed minimal variation in detection outcomes, indicating that ddPCR has relatively low requirements for sample storage duration (**Supplementary Figure 4**).

## Discussion

CAP, one of the most common lower respiratory tract infections, is an important cause of clinical burden and mortality worldwide, with high morbidity and mortality rates in all age groups worldwide (GBD 2019 Diseases and Injuries Collaborators, 2020; Zhang et al., 2023). Bacterial infections remain one of the major causes of morbidity, with *S. pneumoniae, M. pneumoniae and H. influenzae* being the three most prevalent pathogens responsible for CAP, especially *S. pneumoniae* is the leading cause of death from lower respiratory tract infections, according to the Global Burden of Disease Study 2017 (Le Roux and Zar, 2017; GBD 2016 Lower Respiratory Infections Collaborators, 2018). Therefore, early detection of infectious pathogens is crucial for timely clinical diagnosis and to reduce the negative consequences of irrational use of antibiotics (Antimicrobial Resistance Collaborators, 2022). The traditional detection method of bacterial culture is time-consuming, has a low detection rate, and is prone to false negatives (Daxboeck et al., 2003). The application of VITEK MS has improved the sensitivity and specificity of the detection, but the detection process still relies on overnight culture, and relatively pure colonies are obtained before detection, which cannot be directly detected from samples (Cao et al., 2024). In recent years, with the rapid development of molecular diagnostic technology, qPCR has been considered the gold standard for molecular detection, but false-negative results have been obtained for samples with low target concentrations; this depends on the production of a standard curve, which is complicated and time-consuming, and the quality of the standard curve determines the accuracy of the test results (Loens and Ieven, 2016; Sunaga et al., 2020). In addition, some isothermal amplification techniques integrated into microfluidic chips have also been used in recent years for the rapid detection of bacterial infections in clinical settings, but the cost of a single test is relatively high (Feng et al., 2023). Therefore, these tests have not been popularized in large numbers.

The detection of *S. pneumoniae, M. pneumoniae and H. influenzae* DNA in sputum is a rapid diagnostic method for pathogenic bacterial infections (Loens and Ieven, 2016; Sunaga et al., 2020; Cheng et al., 2022; Qiu et al., 2023). ddPCR, a molecular assay that has emerged in recent years, is also used to detect pathogens in different types of clinical samples (blood, sputum, feces, etc.), including *Mycobacterium tuberculosis* (Yang et al., 2017), *Klebsiella pneumoniae* (Feng et al., 2023), Ureaplasma spp (Huang et al., 2021)., hepatitis D virus (Xu et al., 2022), and severe acute respiratory syndrome coronavirus 2 (SARS-CoV-2, Liu et al., 2020). The ddPCR has good sensitivity and specificity. Previous studies have also established qPCR assays for separately detecting these three pathogens (Loens and Ieven, 2016; Sunaga et al., 2020). Compared with qPCR, which quantifies results by a single amplification curve and a CT value, ddPCR assays are performed at the endpoint of the reaction, effectively avoiding sample contaminants such as primer dimers and thus providing more accurate results. Although ddPCR is currently slightly more costly than qPCR, it offers superior performance in certain aspects and remains a critical tool in certain fields, such as ddPCR being clinically important for detecting low abundance targets or analyzing rare mutations (Strain et al., 2013). Previous studies have demonstrated its ability to precisely quantify nucleic acids, such as in HIV viral load monitoring and *Mycobacterium tuberculosis* detection in pleural effusion cfDNA, where ddPCR provides essential guidance for diagnosing infection and assessing patient response to treatment (Strain et al., 2013; Xu et al., 2024). Similarly, the multiple ddPCR method developed in this study was evaluated and shown to be accurate for the quantification of *S. pneumoniae, M. pneumoniae and H. influenzae* in complex samples. At the same time, we also investigated the interference effect of sputum on the two methods. the ddPCR showed less inhibition by the inhibitor in respiratory specimens than the qPCR. Therefore, this method can be used as an effective tool for diagnosis and evaluation of treatment efficacy in patients. In recent years, CRISPR technology has also been used to detect *S. pneumoniae* and *M. pneumoniae*, but the CRISPR method is only qualitative (Qiu et al., 2023; Zhou et al., 2023). In contrast, the ddPCR method enables accurate quantification. Some studies have employed multiple cross displacement ampliflication (MCDA) to detect *H. influenzae*, but this method similarly lacks quantitative capability and is prone to aerosol contamination due to its low reaction temperature (Cheng et al., 2022). In this study, the established multiplex ddPCR method significantly reduces the risk of amplification contamination through droplet generation.

In this study, we established a multiplex ddPCR method for the detection of *S. pneumoniae, M. pneumoniae and H. influenzae* DNA; selected specific genes (the *lytA* gene of *S. pneumoniae*, the *CARDS toxin* gene of *M. pneumoniae* and the specific gene fragment *OmpP6* of *H. influenzae*) as the target sequences for designing specific primers and probes; screened for the optimal primer/probe concentration; and evaluated the sensitivity and specificity of the ddPCR method. The *S. pneumoniae, M. pneumoniae and H. influenzae* ddPCR primers and probes did not cross-react with the other 26 pathogens, indicating that the ddPCR method is highly specific for the detection of *S. pneumoniae, M. pneumoniae and H. influenzae* DNA. The sensitivities of ddPCR for *S. pneumoniae, M. pneumoniae and H. influenzae* DNA were 2.5, 2.8 and 2.0 copies/μL, respectively, which were lower than those of qPCR (27.6, 41.1 and 38.8 copies/μL). In addition, *S. pneumoniae, M. pneumoniae and H. influenzae* were detected in 167 clinical samples and the positive rates of ddPCR were 27.5%, 22.8% and 29.9%, respectively. *M. pneumoniae* and *H. influenzae* were detected simultaneously in 5 samples, *S. pneumoniae* and *H. influenzae* were detected simultaneously in 3 samples, this suggests that bacterial co-infection also exists.

There are also some limitations in this study. First, owing to the number of fluorescence channels and the complexity of the system components, the methods established in this study to detect a small number of bacterial species are limited. These methods are still mainly used for the detection of *S. pneumoniae, M. pneumoniae and H. influenzae*, and three kinds of bacterial cultures do not easily assist in the clinical diagnosis of pathogens. For other common pathogens in lower respiratory tract infection, such as *Klebsiella pneumoniae* (Lin et al., 2015), bacterial cultures can meet the detection needs of bacteria and are therefore not included in probe design. Second, the processing of clinical samples is still cumbersome, and DNA extraction can be further optimized. Notably, some studies have used lysates to extract sputum DNA directly (Cheng et al., 2022). Additionally, the cost of ddPCR tests is higher than that of qPCR tests but much lower than that of second-generation sequencing. In the future, the cost is being gradually reduced through technological iterations such as chip-type droplet generators and high-throughput technologies to facilitate their application in routine detection.

In conclusion, the multiplex ddPCR assay for *S. pneumoniae, M. pneumoniae and H. influenzae* established in this study can accurately detect the bacterial copy number in sputum samples with good sensitivity, specificity and reproducibility. Notably, the ddPCR showed less inhibition by the inhibitor in respiratory specimens than the qPCR. It can be used for the rapid diagnosis of *S. pneumoniae, M. pneumoniae and H. influenzae* infections, providing an adjunctive diagnostic tool for the rapid identification of pathogens causing CAP infection so that patients can receive timely antimicrobial therapy.

## Data Availability

The original contributions presented in the study are included in the article/**Supplementary Material**. Further inquiries can be directed to the corresponding authors.
