# Supplementary material for: Multiplex droplet digital PCR for the detection and quantitation of Streptococcus pneumoniae, Mycoplasma pneumoniae, and Haemophilus influenzae
Source: Front Cell Infect Microbiol. 2025 Jun 20;15:1548492. doi: 10.3389/fcimb.2025.1548492 (PMC12226287; doi:10.3389/fcimb.2025.1548492)
Supplement: Supplementary file 1 [file DataSheet1.docx]

**Multiplex Droplet Digital PCR for the Detection and Quantitation of *Streptococcus pneumoniae*, *Mycoplasma pneumoniae* and *Haemophilus influenzae***

**Supplementary materials:**

**Supplementary Table 1.** Demographic characteristics of patients in this study.

**
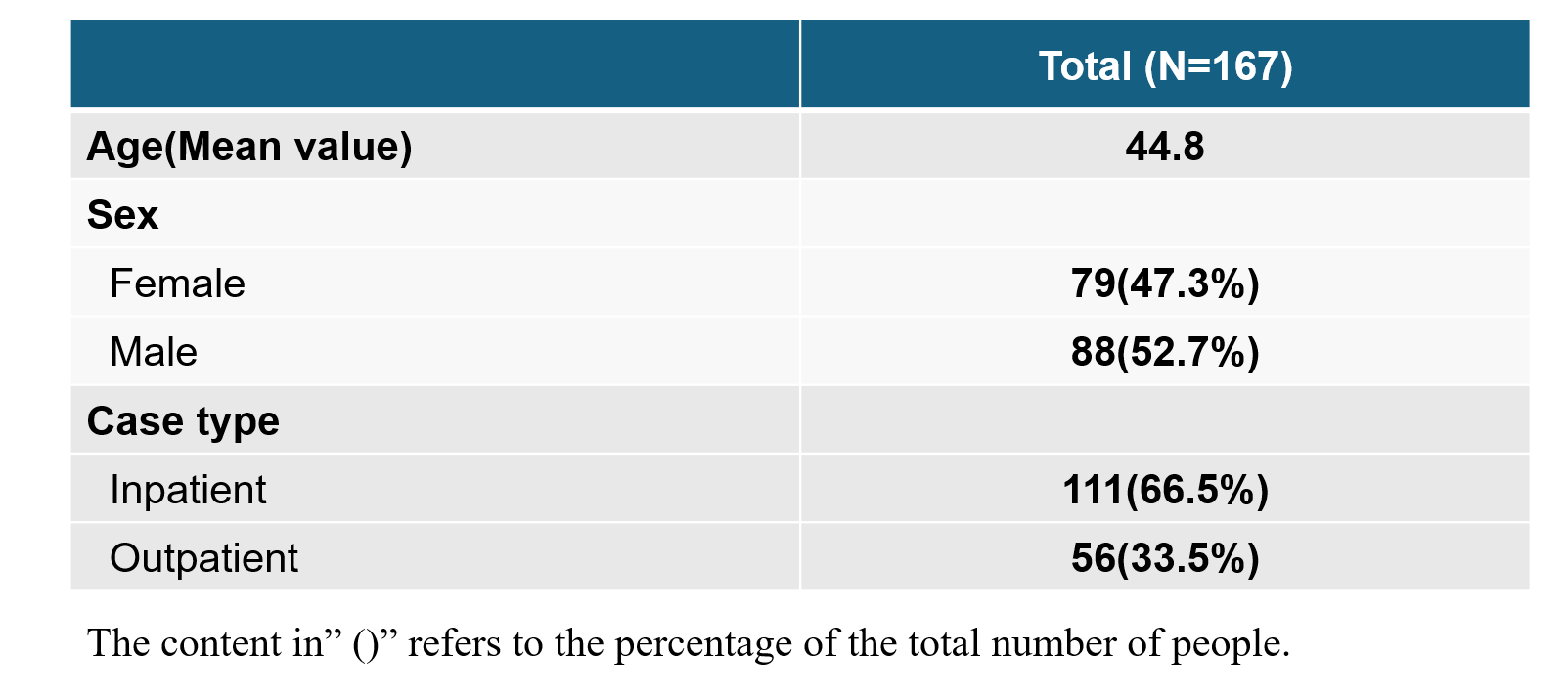
**

**Supplementary Table 2**. Sequences of SP, MP, HI primers.

| name | Sequences（5’-3’） |
| --- | --- |
| SP-F1 | CGCAATCTAGCAGATGAAGCA |
| SP-F2 | CCTTACTTGGCAAAATGGGGC |
| SP-F3 | TGGCAAAATGGGGCATTAGC |
| SP-R1 | GTCAAAGTAGTACCAAGTGCCA |
| SP-R2 | GCGGTCTGCAAGCATATAGC |
| SP-R3 | CTTCCTCCAGCGGTCTGC |
| MP-F1 | GAACCTGGTCATGCTCACCA |
| MP-F2 | GGCTGGTCGTGTTGTAGAGA |
| MP-F3 | CTGGTCATGCTCACCACCC |
| MP-R1 | TGCTAGCGTACTGTGGTAAGG |
| MP-R2 | AGTTCCTTTACACTGCTAGCGT |
| MP-R3 | TCCTTTACACTGCTAGCGTACT |
| HI-F1 | CAACGCCAGCTGCTAAAGTA |
| HI-F2 | TTAAATGCAACGCCAGCTGC |
| HI-F3 | GCAACGCCAGCTGCTAAAG |
| HI-R1 | CAGCATCAACACCTTTACCAGC |
| HI-R2 | AACACTGCACGACGGTTTTT |
| HI-R3 | GCTAACACTGCACGACGGTT |
| SP-Probe | VIC- GCTGGCAGAAGAATGACACTGGCT - BHQ1 |
| MP-Probe | CY5- TCCCCAAGCAGCTTCCGTTGC - BHQ2 |
| HI-Probe | FAM- GGCCAACGTCGTGCAGATGC - BHQ1 |

SP: *Streptococcus pneumoniae*, MP: *Mycoplasma pneumoniae, HI: Haemophilus influenzae.*

**Supplementary Table 3.** The setting of the positive threshold.

|  | positive threshold setting | | |
| --- | --- | --- | --- |
|  | Mean | SD | Mean+3SD |
| SP copy number | 1.07 | 0.96 | 3.9 |
| MP copy number | 0.72 | 0.92 | 3.5 |
| HI copy number | 0.9 | 0.94 | 3.7 |

The test results were repeated 10 times for the negative control. SD: Standard Deviation, CV: Coefficient of Variation.

SP: *Streptococcus pneumoniae*, MP: *Mycoplasma pneumoniae, HI: Haemophilus influenzae.*

**Supplementary Table 4.** Results of ddPCR specificity testing (copy number).

| Strains | *Streptococcus*  *pneumoniae* | | *Mycoplasma*  *pneumoniae* | | *Haemophilus*  *influenzae* | | *Klebsiella*  *pneumoniae* | | *Pseudomonas*  *aeruginosa* | | *Staphylococcus*  *aureus* | |  |
| --- | --- | --- | --- | --- | --- | --- | --- | --- | --- | --- | --- | --- | --- |
| Copy number | 1377.7 | | 1237 | | 25254.4 | | 0 | | 0 | | 0 | |  |
| Strains | *Acinetobacter*  *baumannii* | | *Mycobacterium tuberculosis* | | *Corynebacterium pseudodiphther-iticum* | | *Haemophilus parainfluenzae* | | *Haemophilus*  *lyticus* | | *Haemophilus*  *parahemlyticus* | |  |
| Copy number | 0 | | 0 | | 0 | | 0 | | 0 | | 0 | |  |
| Strains | *Escherichia*  *coli* | | *Candida albicans* | | *Stenotrophomonas maltophilia* | | *Clostridium difficile* | | *Listeria monocytogenes* | | *Streptococcus salivarius* | |  |
| Copy number | 0 | | 0 | | 0 | | 0 | | 0 | | 0 | |  |
| Strains | *Streptococcus mitis* | | *Streptococcus agalactiae* | | *Streptococcus pyogenes* | | *Streptococcus oralis* | | *Streptococcus suis* | | *streptococcus mutans* | |  |
| Copy number | 0 | | 0 | | 0 | | 0 | | 0 | | 0 | |  |
| Strains | *Streptococcus sanguinis* | | *Mycoplasma urealyticum* | | *Mycoplasma hominis* | | *Mycoplasma genitalium* | | *Mycoplasma primatum* | | NC | |  |
| Copy number | | 0 | | 0 | | 0 | | 0 | | 0 | | 0 | |

**Supplementary Table 5.** Comparison of performance between qPCR, ddPCR and chip inspection in detection of SP, HI in 167 clinical samples.

|  | | ddPCR (SP) | | qPCR (SP） | | ddPCR (HI) | | qPCR (HI) | |  |
| --- | --- | --- | --- | --- | --- | --- | --- | --- | --- | --- |
|  |  | Positive | Negative | Positive | Negative | Positive | Negative | Positive | Negative | |
| Microfluidic chip | Postive | 46 | 0 | 43 | 3 | 50 | 0 | 48 | 2 |  |
|  | Negative | 0 | 121 | 0 | 121 | 0 | 117 | 0 | 117 |  |
|  | Total | 46 | 121 | 43 | 124 | 50 | 117 | 48 | 119 |  |

SP: *Streptococcus pneumoniae*, MP: *Mycoplasma pneumoniae*, HI: *Haemophilus influenzae*.

**Target sequence fragments and reference sequences (5’-3’)**

>AP018938.1 *Streptococcus pneumoniae* strain reference sequence

GGGGCGGTTGGAATGCTGAGACCTATGCAGCGGTTGAACTGATTGAAAGCCATTCAACTAAAGAAGAGTTCATGACGGACTACCGCCTTTATATCGAACTCTTACGCAATCTAGCAGATGAAGCAGGTTTGCCGAAAACGCTTGATACAGGGAGTTTAGCTGGAATTAAAACGCACGAGTATTGCACGAATAACCAACCAAACAACCACTCAGACCATGTGGATCCATACCCTTACTTGGCAAAATGGGGCATTAGCCGTGAGCAGTTTAAGCATGATATTGAGAACGGCTTGACGATTGAAACAGGCTGGCAGAAGAATGACACTGGCTACTGGTACGTACATTCAGACGGCTCTTATCCAAAAGACAAGTTTGAGAAAATCAATGGCACTTGGTACTACTTTGACAGTTCAGGCTATATGCTTGCAGACCGCTGGAGGAAGCACACAGACGGCAACTGGTACTGGTTCGACAACTCAGGCGAAATGGCTACAGGCTGG

>LR214945.1 *Mycoplasma pneumoniae* strain reference sequence

TACAATGCCCGCGCCACTGGGGAGAACTTGTTAGATTTAATGCGTCAAAGACAAGTAGTATTTGACTCTGGTGATCGAGAAATGGCACAAATGGGAATTAGAGCTTTACGCACTTCCTTTGCGTATCAACGTGAATGGTTTACCGATGGTCCAATTGCAGCAGCTAATGTCCGTAGTGCTTGACTAGTAGATGCTGTTCCCGTTGAACCTGGTCATGCTCACCACCCGGCTGGTCGTGTTGTAGAGACTACTAGAATTAATGAACCGGAAATGCACAACCCTCATTATCAAGAGCTGCAAACCCAAGCCAATGATCAACCATGATTGCCAACACCAGGAATAGCTACTCCTGTACATTTATCAATTCCCCAAGCAGCTTCCGTTGCTGATGTTTCGGAAGGTACTTCCGCTTCGCTATCGTTTGCGTGCCCTGATTGAAGTCCACCTTCTAGTAATGGTGAAAATCCGCTAGACAAATGCATTGCGGAAAAGATTGATAACTATAACCTACAATCCTTACCACAGTACGCTAGCAGTGTAAAGGAACTGGAAGATACACCAGTATACCTAAGGGGAA

>KC332053.1 *Haemophilus influenzae* strain outer membrane protein P6 reference sequence

TTAGCAGCTTGTAGTTCATCTAACAACGATGCTGCAGGCAATGGTGTTGCTCAAACTTTTGGCGGTTACTCTGTTGCTGATCTTCAACAACGTTACAATACCGTTTATTTCGGTTTTGATAAATATGACATTACTGGTGAATACGTTCAAATCTTAGATGCGCACGCTGCATATTTAAATGCAACGCCAGCTGCTAAAGTATTAGTAGAAGGTAACACTGATGAACGTGGTACACCAGAATACAACATCGCATTAGGCCAACGTCGTGCAGATGCAGTTAAAGGTTATTTAGCTGGTAAAGGTGTTGATGCTGGTAAATTAGGCACAGTATCTTACGGTGAAGAAAAACCTGCAGTATTAGGTCATGATGAAGCTGCATATTCTAAAAACCGTCGTGCAGTGTTAGCGTACTAA


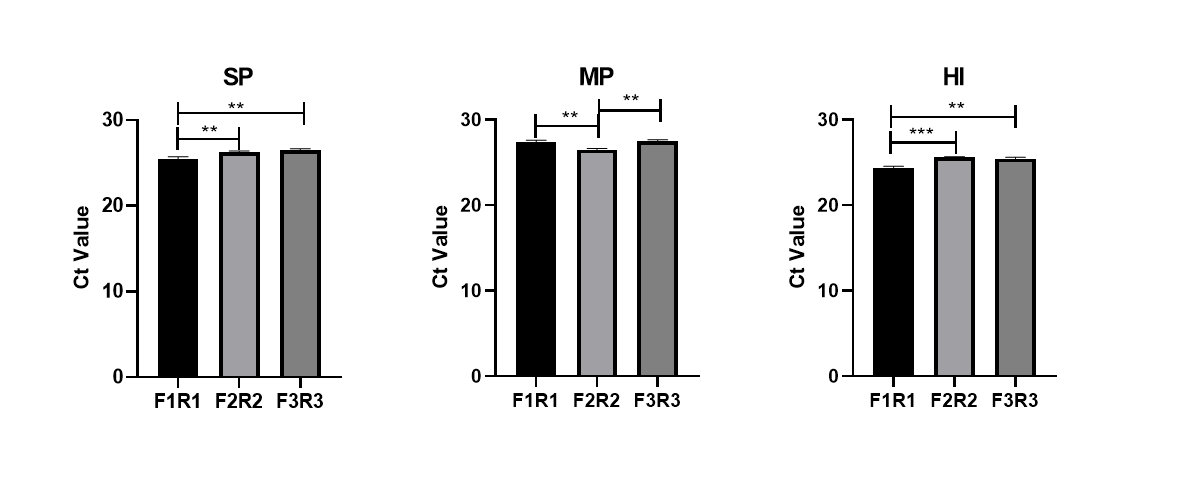


**Supplementary Figure 1**

The optimal primers were selected by comparing the CT values of different primers in single qPCR. SP: *Streptococcus pneumoniae*, MP: *Mycoplasma pneumoniae, HI: Haemophilus influenzae.*

*
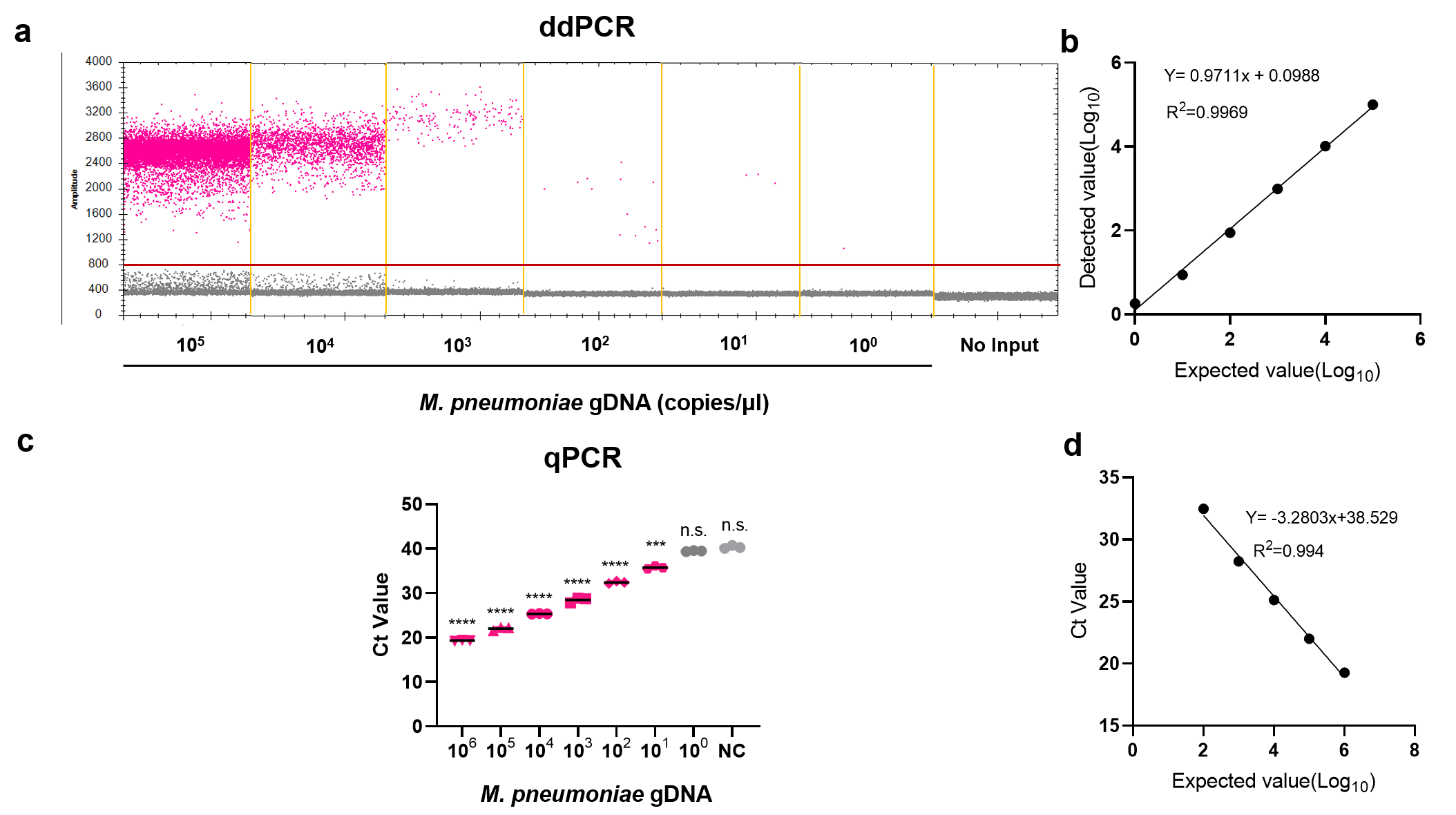
*

**Supplementary Figure 2**

The dynamic range of the ddPCR assay to detect *M. pneumoniae using* Reference strain. (a)The 10-fold serial dilution of *M. pneumoniae* gDNA were detected by the ddPCR; the pink points represent the positive signal; Correlation analysis to determine the dynamic detection range. (b) The expected values (converted to log10) of *M. pneumoniae* gDNA, were plotted on the Y axis and ddPCR detected values (converted to log10) on the X axis to perform linear analysis. Data are representative of at least three repeated experiments for different concentrations of template DNA. (c) The 10-fold serial dilution of *M. pneumoniae* gDNA were detected by the qPCR; (d)The expected values (converted to log10) of *M. pneumoniae* gDNA were plotted on the Y axis and qPCR detected values (converted to log10) on the X axis to perform linear analysis. DNase/RNase-free water was used as the negative control. The experiment was repeated three times (means ± SD). ***P < 0.001, ****P < 0.0001 and n.s. indicates no statistically significant difference.

.

*
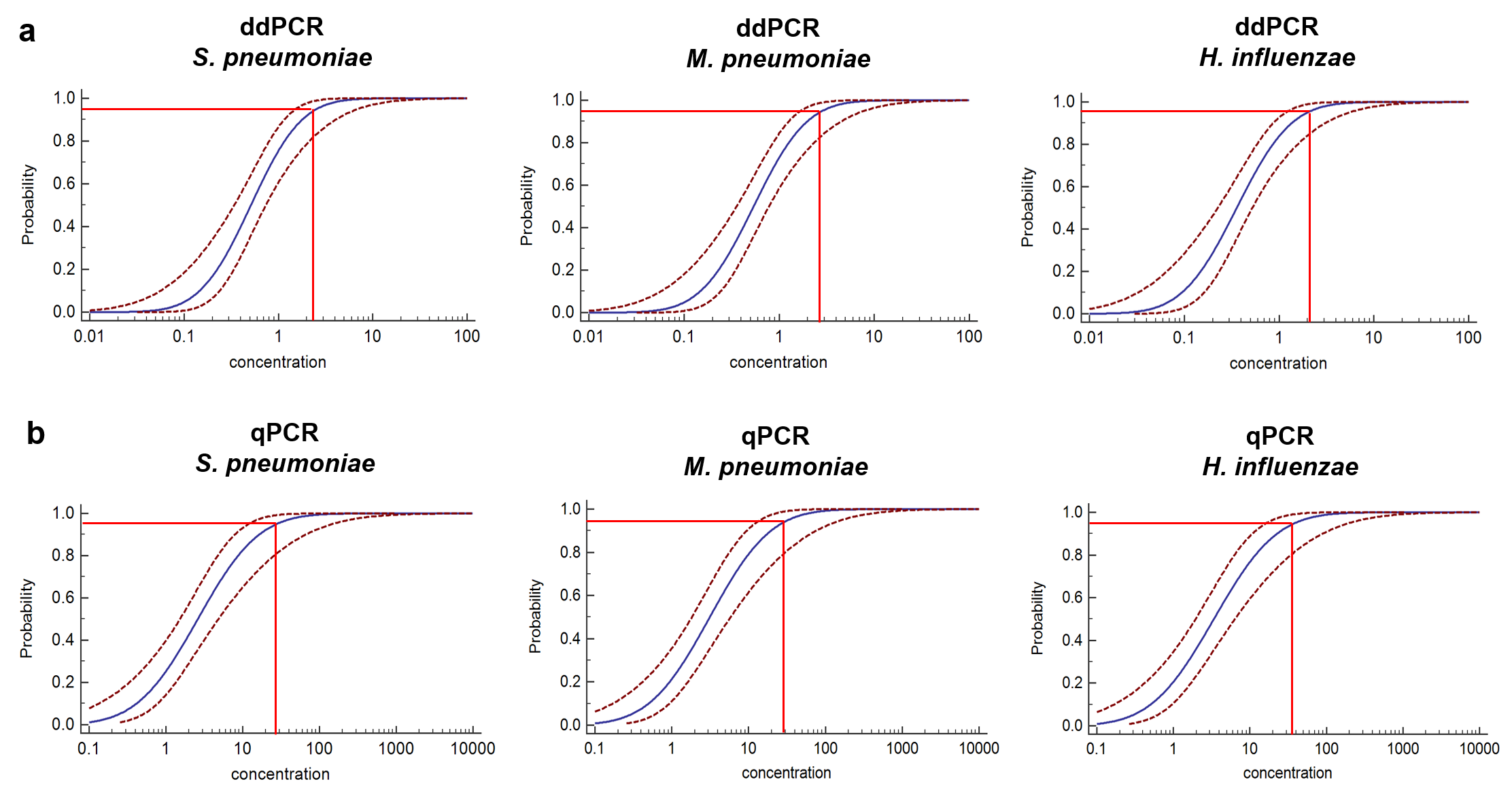
*

**Supplementary Figure 3.** Determining the LoD of the ddPCR assay.

(a) The LoD for *S. pneumoniae*, *M. pneumoniae*, and *H. influenzae* in the multiplex qPCR assay was determined using probit analysis of sigmoid curves. Repeated ddPCR assays with different concentrations of *S. pneumoniae*, *M. pneumoniae* and *H. influenzae* gDNA were performed near the detection limits determined in the pre-experiment. The X-axis represents the expected concentration, and the Y-axis represents the proportion of positive results in the same experiment. The blue line is the probit curve, and the red dashed line are 95% confidence interval (95% CI), the experiment was repeated twenty times for each concentration in the same parallel reactions. (b) The probit analysis sigmoid curve was used to determine the LoD of the multiplex qPCR for *S. pneumoniae*, *M. pneumoniae* and *H. influenzae* detection. The analytical method was the same as in (a). The experiment was repeated twenty times for each concentration in the same parallel reactions.

*
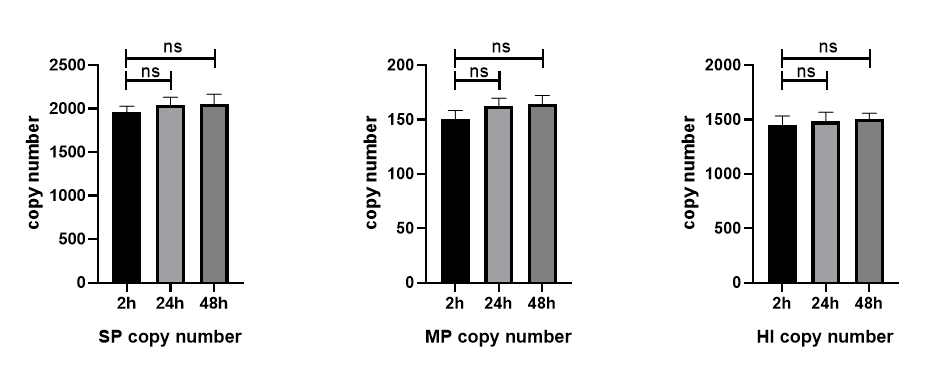
*

**Supplementary Figure 4.** The ddPCR detection results of samples with different storage times. SP: *Streptococcus pneumoniae*, MP: *Mycoplasma pneumoniae, HI: Haemophilus influenzae.*
